# Stop CRYing! Inhibition of cryptochrome function by small proteins

**DOI:** 10.1042/BST20190062

**Published:** 2022-03-21

**Authors:** Valdeko Kruusvee, Arendse Maria Toft, Blanche Aguida, Margaret Ahmad, Stephan Wenkel

**Affiliations:** 1 Department of Plant and Environmental Sciences, University of Copenhagen, Thorvaldsensvej 40, 1871 Frederiksberg C, Denmark; 2 Copenhagen Plant Science Centre, University of Copenhagen, Thorvaldsensvej 40, 1871 Frederiksberg C, Denmark; 3 Sorbonne Universités –CNRS, UMR8256 – IBPS, Photobiology Research Group, Paris, France; 4 NovoCrops Centre, University of Copenhagen, Thorvaldsensvej 40, 1871 Frederiksberg C, Denmark

**Keywords:** cryptochrome, light signaling, microproteins

## Abstract

Plants can detect the presence of light using specialised photoreceptor proteins. These photoreceptors measure the intensity of light, but they can also respond to different spectra of light and thus ‘see' different colours. Cryptochromes, which are also present in animals, are flavin-based photoreceptors that enable plants to detect blue and ultraviolet-A (UV-A) light. In Arabidopsis, there are two cryptochromes, CRYPTOCHROME 1 (CRY1) and CRYPTOCHROME 2 (CRY2) with known sensory roles. They function in various processes such as blue-light mediated inhibition of hypocotyl elongation, photoperiodic promotion of floral initiation, cotyledon expansion, anthocyanin production, and magnetoreception, to name a few. In the dark, the cryptochromes are in an inactive monomeric state and undergo photochemical and conformational change in response to illumination. This results in flavin reduction, oligomerisation, and the formation of the ‘cryptochrome complexome'. Mechanisms of cryptochrome activation and signalling have been extensively studied and found to be conserved across phylogenetic lines. In this review, we will therefore focus on a far lesser-known mechanism of regulation that is unique to plant cryptochromes. This involves inhibition of cryptochrome activity by small proteins that prevent its dimerisation in response to light. The resulting inhibition of function cause profound alterations in economically important traits such as plant growth, flowering, and fruit production. This review will describe the known mechanisms of cryptochrome activation and signalling in the context of their modulation by these endogenous and artificial small inhibitor proteins. Promising new applications for biotechnological and agricultural applications will be discussed.

## Cryptochromes are blue light photoreceptors

**Figure 1.**
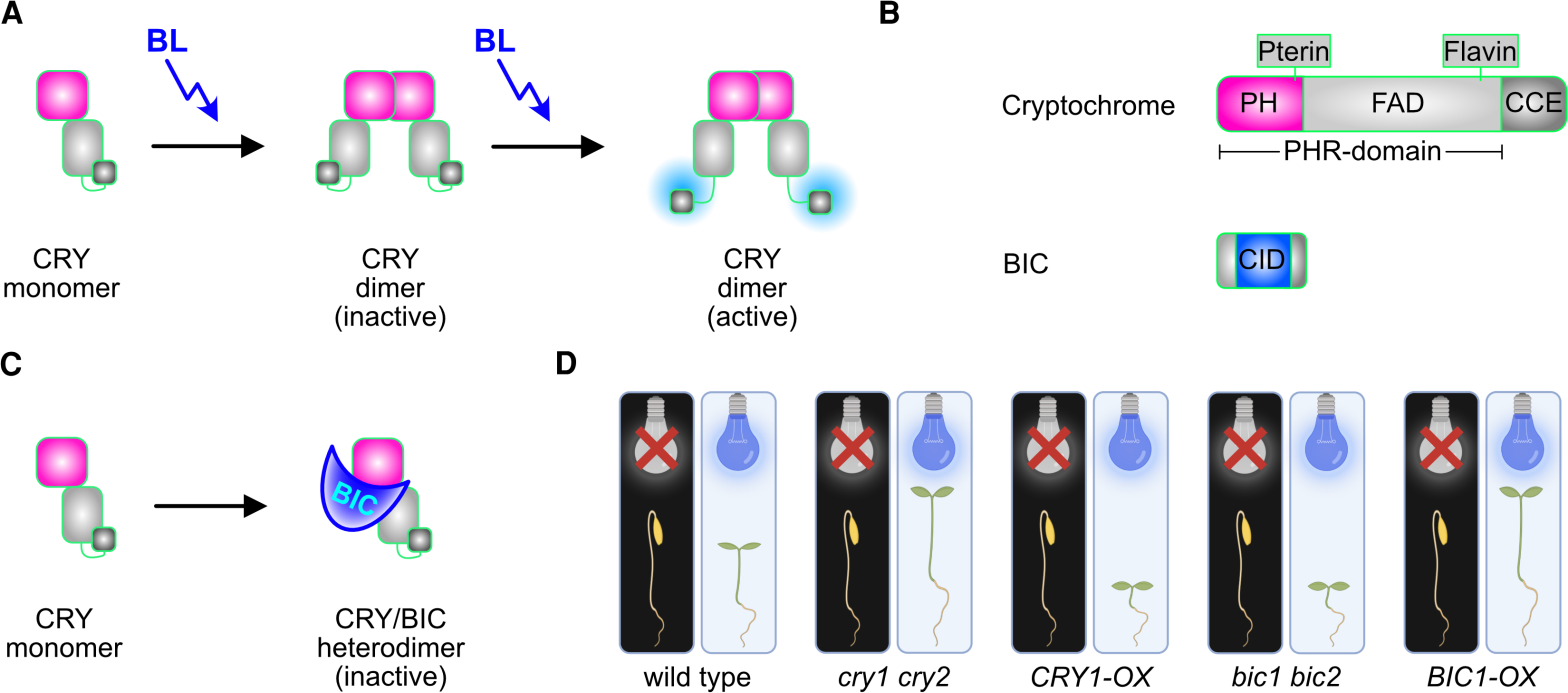
Control of cryptochromes by BICs. (**A**) In response to blue light (BL), the CRY monomers undergo photochemical reduction in the flavin cofactor (FAD), followed by dimerisation and formation of an active dimer. (**B**) Domain overview of cryptochromes and BICs. (**C**) The CRY PH domain can interact with the BIC CID domain, forming an inactive CRY-BIC heterodimer. This prevents the homodimerisation and subsequent activation of CRYs. (**D**) In wild-type plants (first panel), exposure to blue light triggers the de-etiolation process and stops the hypocotyl from elongating. The cry1/cry2 double mutant (second panel) leads to elongated hypocotyls, while the overexpression of CRY1 (third panel) results in shortened hypocotyls. The bic1/bic2 double mutant (fourth panel), which is unable to repress cryptochrome function, has a phenotype similar to the CRY1 overexpression mutant, while the overexpression of BIC1 (fifth panel) has a similar phenotype to the cry1/cry2 double mutant.

Cryptochromes (CRYs) function as light-activated relays that allow plants to switch between light (active) and dark (inactive) biological states. In response to blue light the flavin cofactor first undergoes photochemical reduction [1,2] followed by conformational change and dimerisation to the biologically active signalling state (Figure 1A). CRYs are flavoproteins with structural similarities to photolyases from which they evolved. However, unlike photolyases that repair DNA damage resulting from exposure to ultraviolet light, most CRYs are not involved in DNA repair. In *Arabidopsis thaliana*, three CRYs have been reported: cryptochrome 1 (CRY1), cryptochrome 2 (CRY2), and cryptochrome 3 (CRY3). CRY1 and CRY2, the focus of this review, have largely overlapping functions in photomorphogenesis, cotyledon expansion, stomatal development, and the production of anthocyanins [3–7]. CRY1 is stably expressed in the light and regulates plant growth and development at higher light intensity [8,9]. CRY2, which is degraded upon exposure to blue light, has a more specialised role under conditions of limiting blue light intensity, as well as in the photoperiod promotion of floral initiation [3]. CRY1 is located in both the nucleus and cytoplasm, while CRY2 is nuclear-localised. CRY3, which localises to the chloroplast and mitochondria, is part of the CRY-DASH (cryptochrome-Drosophila, Arabidopsis, Synechocystis, human) class of cryptochromes and has been reported to possess the ability to repair cyclobutane pyrimidine dimers (CPDs) in UV-damaged single-stranded DNA [10–12]. Cryptochromes have two domains: the highly conserved amino-terminal photolyase homologous region (PHR) domain to which the FAD cofactor is bound, and the CCE-domain which is a C- terminal extension of variable length and which is poorly conserved among cryptochromes (Figure 1B). The FAD-binding domain non-covalently binds the catalytic chromophore flavin-adenine dinucleotide (FAD). In photolyases, the PHR domain binds a second chromophore, pterin (methenyltetrahydrofolate; MTHF), however this chromophore is absent from the purified fractions of plant cryptochromes CRY1 and CRY2.

Various processes such as phosphorylation, ubiquitination, and the interaction with regulatory proteins regulate the activity of cryptochromes, but the major factor that influences plant cryptochrome activity is light. Details of the cryptochrome photochemical response to blue light have been extensively studied and are well characterised by a variety of biophysical and spectroscopic techniques [2]. To explain the signalling reaction, cryptochromes are shown to exist as inactive monomers in the dark with the PHR and CCE domain folded in an inaccessible form; in this state, the FAD cofactor is fully oxidised [1,13]. Upon photoexcitation, the oxidised FAD becomes semi-reduced, triggering conformational changes that enable various processes such as the homo-oligomerisation, phosphorylation, and ubiquitination of CRYs. These conformational changes also result in exposure of the nuclear localisation signals located on the CRY C-terminal domains and provide accessibility for the signalling partners that interact with CRYs, in this way initiating the many different signalling pathways in which CRYs are implicated [14–17]. Recent electron microscopy studies of CRY2 have revealed that blue light irradiation triggers tetramerisation of CRY2 through a network of hydrophobic interactions and hydrogen-bonding interaction, and that FAD photoreduction is necessary for this to occur [18].

## Inhibition of CRY function through specific protein inhibitors

The PHR domain of CRY1 and CRY2, also referred to as CNT1 and CNT2 respectively, are required for the function of CRYs and for mediating CRY signalling [19]. In fact, the simple addition of a nuclear localisation signal to the CNT1 domain was sufficient to confer Cry biological activity to overexpressing plants, including short hypocotyls in blue light. Deletion and point mutations in CNT1, however, can result in the abolition of CRY1 oligomerisation and subsequent biological activity. The overexpression of such point mutants in the *cry1* mutant background results in photomorphogenic phenotypes resembling *cry1* mutants that develop long hypocotyls that are unresponsive to blue light [17]. In addition, the overexpression of CNT1 lacking the nuclear localisation signal in a wild-type genetic background results in transgenic plants with dominant-negative phenotypes consisting of unexpanded and long hypocotyls in the presence of blue light. This may be because of titration effects of CNT1 on endogenous CRY1 leading to the formation of inactive heterodimers [17,20].

In an activation-tagging screen in Arabidopsis, two small and related proteins named BLUE LIGHT INHIBITOR OF CRYPTOCHROMES (BIC1 and BIC2) were identified [21]. Overexpression of BICs disrupts cryptochrome dimerisation and the formation of nuclear photobodies, and transgenic plants resemble *cry1 cry2* double mutants. Conversely, *bic1 bic2* double mutant plants resemble plants with elevated cryptochrome activity (Figure 1C,D). Moreover, it was shown that cryptochromes positively regulate the transcription of both *BIC* genes in response to blue light [22]. Thus, BICs establish a negative feedback mechanism that down-regulate CRY activity under conditions of continuous high light, and likely has adaptive significance. Both BICs encode proteins with no defined protein functional domains.

## Evolution of plant cryptochrome inhibitors — a unique regulatory mechanism

**Figure 2.**
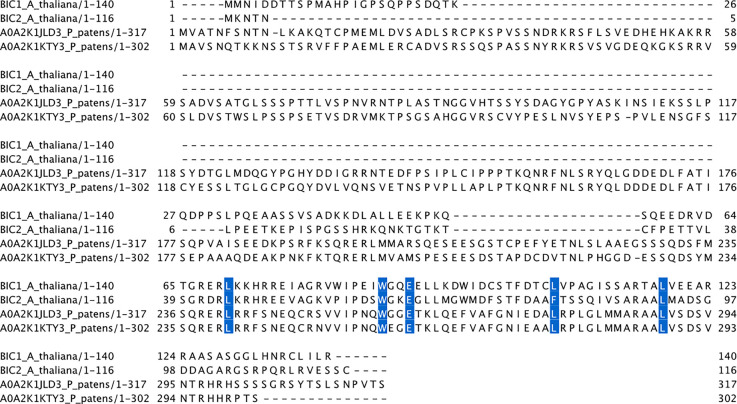
Multiple sequence alignment of *A. thaliana* BIC1 and BIC2 CID domain with *P. patens* BIC homologues. Residues highlighted in blue have been shown to abolish the binding of BIC2 to CRY2 [18].

While cryptochromes are found in both plants and animals, BIC-like mechanisms that modulate cryptochrome function by preventing oligomerisation are only found in land plants. The origin of BICs is unclear, although based on the lack of homologous proteins in other kingdoms of life it is likely that they initially evolved from an early land plant ancestor. The earliest land plant known to harbour BICs is *Physcomitrium patens*, a bryophyte model organism. These *Pp*BIC homologues show a high level of sequence conservation with Arabidopsis BICs in the domain that interacts with cryptochromes. In particular, the residues that, when mutated, abolish binding between CRY2 and BIC2 [18] are almost invariably conserved between the *P. patens* and *A. thaliana* BICs (Figure 2). Unlike almost all other BIC homologues, including those of Arabidopsis, the *P. patens* BICs contain much longer N-terminal extensions. It is unclear whether these sequences have any function in *P. patens*, although these regions have no detectable homologues in any other organisms. Equally, the protein annotations for these sequences are that of a disordered region. It is possible that these disordered regions represent novel interaction domains specific to bryophytes or indeed to *P. patens*, although their interaction targets, if any, are currently unknown. It is also possible that these regions were acquired when *P. patens* underwent a whole genome duplication (WGD) event in its evolution [23] and have not yet undergone evolutionary trimming.

**Figure 3.**
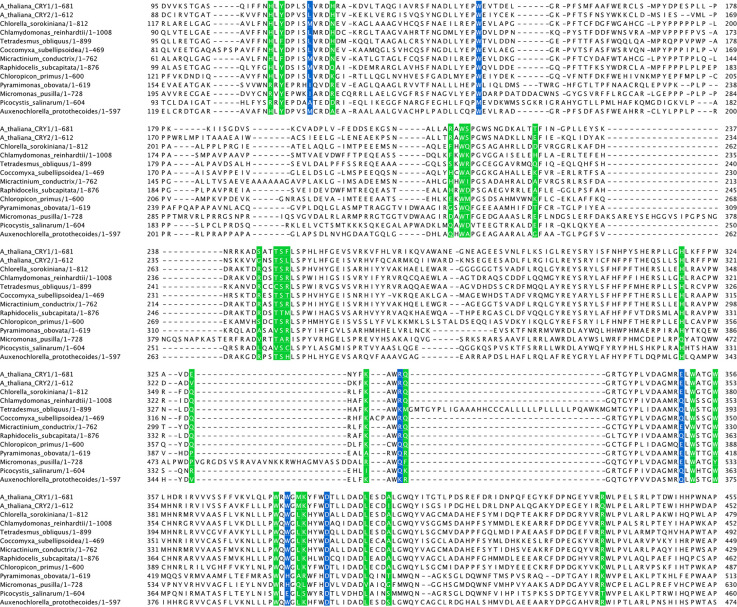
Multiple sequence alignment of *A. thaliana* CRY1 and CRY2 homologues. In green and blue are all CRY2 residues that interact with BIC2 (according to [18]). Those in blue were shown by mutational studies to abolish or reduce binding of BIC2 to CRY2.

A clue to the evolutionary origin of BICs may lie in the green algae. They contain cryptochromes but unlike in land plants they have no detectable BICs. Analysis of their cryptochromes reveals that many of the amino acid residues involved in contacting BICs in land plants are not conserved, although not all of them affect the binding between CRY2 and BIC2 [18] (Figure 3). However, not all the potential interaction residues were addressed by mutational studies, so it is quite likely that other amino acids contribute to the interaction between BIC2 and CRY2. In addition, L109 is variable between *A. thaliana* and green algae BICs. In the analysed green algae CRYs, that position has either leucine, methionine, isoleucine, or alanine. The L109A mutation abolished the interaction between BIC2 and CRY2 [18], and at least one green algae CRY2 possesses that mutation. In addition, the L109 in *A. thaliana* CRY2 forms a part of a hydrophobic interface with BIC2. It is likely that a methionine in that position, which is seen in the green algae homologues, would abolish BIC2 binding due to the much larger sidechain. It is unknown whether a L109I mutation would be neutral, although it may be possible that while the mutation does not abolish binding, it may alter the binding kinetics. In addition, the residues involved in binding the FAD cofactor are almost invariably conserved between the green algae and *A. thaliana* cryptochromes. This suggests that the interaction surface for BICs evolved on the cryptochromes without disrupting their overall fold or function. Given the absence of compatible BIC interaction residues in these green algae cryptochromes, it is unlikely that these species possess unidentified BIC homologues. In addition, the classical plant cryptochromes have only been found in some algal species [24]. As such, the ancestor of BICs most likely evolved in an ancient ancestor of land plants after a WGD event.

The diversification of protein–protein interaction (PPI) networks after WGD has recently been demonstrated using SEPALLATA3 (SEP3), a MADS-box protein transcription factor. In the study, the SEP3 ancestor ancE was shown to act as a PPI hub, being able to bind all MADS-box proteins. After a WGD event, the duplicated ancE genes evolved further and generated novel PPI interfaces, losing and gaining protein interaction partners in the process [25]. A similar event may have occurred in this unknown ancestor where after a WGD a BIC-like protein was created through chromosomal reorganisation. If this BIC ancestor was similar to modern BICs, it would have been largely unstructured. This structural and conformational freedom may have enabled it to interact (weakly) with cryptochromes. It has furthermore been shown that the C-terminal intrinsically disordered tails of cryptochromes serve as autoinhibitory regions in many organisms [26], including in Arabidopsis. If BIC ancestors could compete with these autoinhibitory regions to impart a novel regulatory function, they could allow for a more finely tuned control of the blue light response, and the circadian clock and flowering time regulation. As the sessile plants have little influence on the environment they are exposed to, this response may have proved advantageous and was subsequently maintained.

## How are all the CRYs and BICs organised?

Whole genome duplication (WGD) events are much more common in plants than in other eukaryotes [27]. After a WGD event, the subsequent fate of the duplicated genes can lead to sub-functionalisation where the two copies divide the responsibilities of the original gene between them; neo-functionalisation where one of the genes incurs progressive mutations that changes, for example, their function or tissue expression patterns; or a loss of one of the duplicated genes to return to original monogenic state. Generally, the resulting duplicated genome is eventually lost and the remaining regions are reorganised to reinstate the diploid status, although some duplicated genes escape this fate [28].

As BICs control the activity of CRYs, it would be reasonable to assume that an increase in the number of CRY homologues in a genome would also result in an increase in the number of BIC homologues, and *vice versa*. Given the relatively large number of known WGDs that have occurred in the past ∼100–150 million years [29], we investigated how this has affected the copy numbers of BICs and cryptochromes in different species.

Using the *Arabidopsis thaliana* BIC1 (UniProt accession Q9LXJ1) and CRY1 (UniProt accession Q43125) sequences, we retrieved all homologues of CRYs and BICs in selected land plants (Table 1). One of the surprising discoveries was the relatively consistent low number of BIC homologues even in species that have undergone recent WGDs. There have been two WGDs in the Brassicaceae family and one in the Brassica genus within the last ∼80 million years. However, both the diploid *Arabidopsis thaliana* and *Brassica oleracea* have only two BIC homologues each, while the tetraploid *Brassica napus* has five. Even more strikingly, *Zea mays* which underwent the most recent WGD in the last 12–15 million years [30], as well as two WGDs in its ancestral lineage ∼100 million years ago, contains only three detectable BIC homologues. Surprisingly, even the hexaploid common wheat (*Triticum aestivum*) only contains three known BICs.

**Table 1:** Numbers of BICs and CRYs in selected organisms

Organism	BICs	Cryptochromes
*Arabidopsis thaliana*	2	4
*Brachypodium distachyon*	2	5
*Brassica napus*	5	13
*Brassica oleracea var. olerace*	2	7
*Cucumis sativus*	3	3
*Glycine max*	7	9
*Hordeum vulgare subsp. vulgare*	2	5
*Medicago truncatula*	3	5
*Oryza sativa subsp. japonica*	2	5
Physcomitrium patens*	2	5
*Solanum lycopersicum*	2	5
*Solanum tuberosum*	3	4
*Sorghum bicolour*	2	5
*Triticum aestivum*	3	14
*Zea mays*	3	10
*Populus trichocarpa*	6	6

*Weak homology

On the other hand, soybean (*Glycine max*) which also underwent a WGD relatively recently (∼13 million years ago [31]) contains seven BIC homologues, highest of all analysed plant species. We hypothesise that the large number of BICs is the result of the relatively recent WGD, and that there is significant evolutionary pressure to lose these extra copies. On the other hand, the black cottonwood (*Populus trichocarpa*) which had the most recent WGD event ∼58 million years ago [32], still contains six BIC homologues [33]. This is in contrast with other analysed species that have undergone WGD event(s) in more recent history and have lost the extra copies. It is unclear why black cottonwood species has retained such a large number of BICs, although it has been suggested the molecular clock of the black cottonwood is much slower than in other species that may contribute to the slow loss of these extra copies [33].

The number of cryptochrome genes in these species are approximately twice as large as the number of BICs, but due to lack of experimental evidence it is currently unknown how many of these cryptochromes are actual targets of BICs. Three notable exceptions are the common wheat, which has 14 cryptochromes compared with its three BICs; and maize with ten cryptochromes and three BICs, as well as the black cottonwood, which contains an equal number of BICs, and cryptochromes. It is also not known whether all these BIC and CRY proteins are functional, or the processes in which they are implicated. Given the large number of homologues in some species, these extra copies do not seem to be deleterious. It is possible that either the extra copies of CRYs and BICs balance each other out, some of the homologues are not expressed, or that the increased CRY and BIC protein levels do not negatively affect the plant growth. Taken together, these observations seem to suggest that extra copies of BICs are lost quicker from respective genomes than the cryptochromes; and that generally plants drift towards having between two to three BICs and four to five cryptochromes.

BICs provide an extra level of regulation of the CRY receptor to light intensity. In particular, it might be evolutionarily advantageous to have a receptor that responds better to low intensity blue light (where limiting light signals need to be maximised) than to high intensity blue light (where the light signal is saturating). The fact that BICs accumulate at high light intensities allows the system to be less active at a higher light intensity, where light intensity is saturating, as compared with the lower light intensity, where maximal light capture is desired.

## Synthetic biology approaches

Optogenetics combines genetics and optical methods to control biological processes in living cells by light. The field is founded on the discovery of channel rhodopsins, which are light-gated cation channels [34]. More recent advances also make use of plant photoreceptors to control biological processes in both plants and animal cells with light [35]. Cryptochromes have also been employed as optogenetic tools. Here, the ability of Arabidopsis CRY2 to interact with the basic helix-loop-helix transcription factor CIB1 [36] has been exploited to reversibly control protein translocation, transcriptional activation and recombination in mammalian cells [37]. To create more complex and feedback-regulated signalling pathways, BICs could be added as relays in respective synthetic optogenetic pathways. Such a feedback relay would be entirely post-transcriptional and could thereby provide considerable added fine-tuning of the response to light.

In addition to BICs, cryptochrome activity can be regulated by synthetic microProteins. MicroProteins are small, single-domain proteins that act in a dominant-negative manner on target proteins they are related to [38]. They generally exist as individual genes, although small gene families of microProteins are known. A hallmark of microProteins is that they engage in protein–protein interactions (PPIs) either with their targets directly, or with the proteins that interact with their target. MicroProteins are thought to have evolved from larger ancestral proteins by losing functional domains, retaining only the PPI domains. Diverse developmental processes such as leaf development [39,40], shoot branching [41] and flowering time control [42] have been shown to be modulated by microProteins.

**Figure 4.**
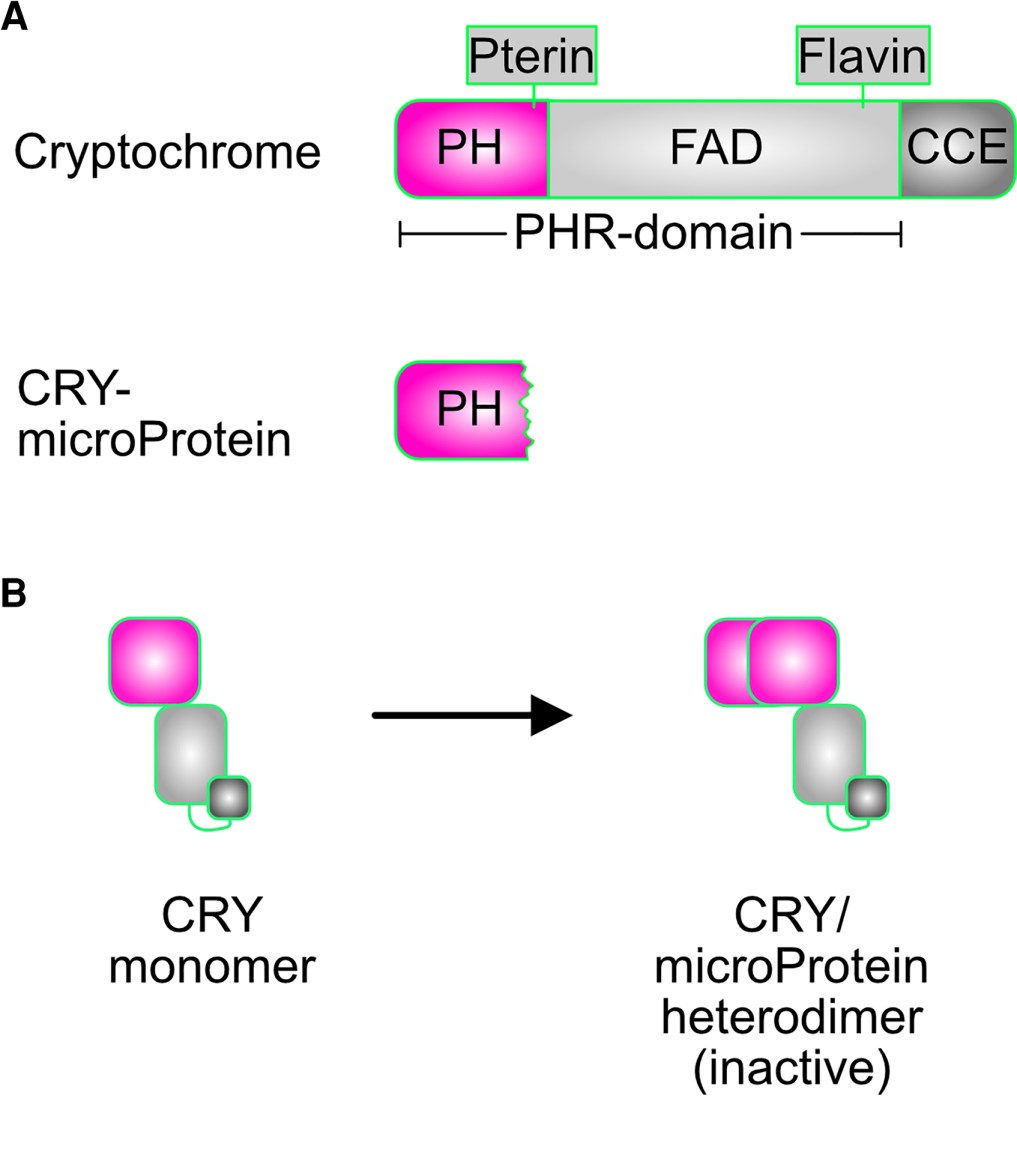
Synthetic microProteins can control cryptochrome function. (**A**) Domain overview of full-length *A. thaliana* CRY1 (top) and the synthetic CRY-microProtein that contains only the PH domain required for dimerisation. (**B**) The CRY-microProtein disrupts the formation of an active CRY homodimer by forming an inactive CRY-microProtein/CRY heterodimer, thereby sequestering the CRY monomers.

In a proof-of-concept study that microProteins can be designed to interfere with known signalling proteins, microProteins related to CRY1 were generated [43]. For this synthetic microProtein approach, the photolyase (PHR) domain of Arabidopsis CRY1 was used (Figure 4A). This CRY1-microProtein was able to physically interact with the full-length CRY1 protein in yeast, and overexpression of CRY1-microProtein in transgenic plants elicited strong changes in the response to blue light. Specifically, CRY1-microProtein overexpression plants strongly resembled *cry1 cry2* double mutant plants displaying elongated hypocotyls when grown in blue light conditions. It is likely that the CRY1-microProtein inactivates CRY1/2 by preventing the formation of functional cryptochrome homodimers (Figure 4B). Although their size and dominant-negative mode of action make BICs similar to microProteins, their lack of sequence relatedness with a larger multi-domain protein excludes BICs from being classified as genuine microProteins.

Whether these synthetic microProteins also interfere with BIC1/2 function is currently not clear, although in principle it should be possible to create novel CRY-derived synthetic microProteins that target the BICs rather than the cryptochromes themselves. This would allow the targeting of cryptochrome function at many levels including by directly interfering with their dimerisation activity, or indirectly by controlling the activity of BICs. Together, these small protein inhibitors could be used in combination to alter the blue-light response in plants to bring about desired changes such as flowering or fruiting. Alternatively, they could be combined with existing optogenetic tools to allow for a more fine-tuned spatiotemporal control over proteins *in vivo*and *ex vivo*.

## Concluding remarks

In spite of cryptochromes being an important class of blue-light sensing photoproteins that are necessary in triggering fundamental developmental stages such as (de)etiolation and floral initiation, we are still learning about their function and regulation. The dimerisation activity of cryptochromes, which is essential for their biological function, can be regulated using small endogenous and synthetic proteins. These proteins could be utilised as on/off switches for cryptochrome signalling and therefore regulate every aspect of plant development under the control of blue light. This would be especially important in areas such as (vertical) farming by allowing direct control over when or how fast the plants grow, flower, or even produce nutrients. Developmental changes such as flowering could be delayed or induced during (dis)advantageous environmental conditions, allowing growers to maximise yields and minimise losses. Equally, the discovery of cryptochrome regulators that function in the inactive (dark) state could be incorporated into the existing cryptochrome-based optogenetic tools. This would allow researchers to both activate and inactivate protein–protein interactions in response to blue light. Taken together, cryptochromes and their regulators provide an exciting and powerful approach to (plant) biotechnological engineering.

## Perspectives

- Cryptochromes are blue light receptors found in plants and animals which regulate many important cellular functions like the circadian clock, growth, and development. There are also many optogenetic tools that have been developed using cryptochromes.
- Cryptochromes are regulated by light, magnetic fields, and temperature through induction of conformational change and posttranslational modifications including phosphorylation and ubiquitination. Plant cryptochromes physiological function can further be modulated by small-molecule inhibitor proteins (microproteins).
- Better understanding of how small protein inhibitors of Cry function should provide new insight into how cryptochromes can be regulated in plants as well as help in development of next-generation optogenetic tools.

## Abbreviations

CRYs: cryptochromes
FAD: flavin-adenine dinucleotide
PHR: photolyase homologous region
PPI: protein–protein interaction
SEP3: SEPALLATA3
WGD: whole genome duplication
